# 人非小细胞肺癌NCI-H1299细胞特异性结合肽的筛选及鉴定

**DOI:** 10.3779/j.issn.1009-3419.2020.103.17

**Published:** 2020-12-20

**Authors:** 琪 左, 超 郭, 卫平 樊, 晓峰 杨, 帆 张

**Affiliations:** 1 030000 太原，山西医科大学微生物学与免疫学教研室 Department of Microbiology and Immunology, Shanxi Medical University, Taiyuan 030000, China; 2 030000 太原，山西医科大学第一医院泌尿外科 Department of Urology, the First Hospital of Shanxi Medical University, Taiyuan 030000, China

**Keywords:** 噬菌体展示技术, 肺肿瘤, NCI-H1299细胞, 特异性结合肽, Phage display technology, Lung neoplasms, NCI-H1299 cells, Specific binding peptide

## Abstract

**背景与目的:**

非小细胞肺癌（non-small cell lung cancer, NSCLC）是最常见的肺癌组织学类型，也是病死率最高的恶性肿瘤之一。多肽作为光学分子成像探针的主体可以实现肿瘤的早期筛查及诊断，提高患者存活率。本研究旨在利用体内噬菌体展示技术筛选与人NSCLC细胞NCI-H1299高度结合的小分子多肽并通过体外实验鉴定其结合特异性。

**方法:**

制备NCI-H1299细胞荷瘤裸鼠模型，用噬菌体展示环七肽库进行3轮体内筛选后随机挑取噬菌体克隆，免疫组织化学法及酶联免疫吸附法（enzyme-linked immunosorbent assay, ELISA）鉴定噬菌体克隆对NCI-H1299细胞的亲和力。提取阳性单克隆噬菌体DNA测序获得外源多肽氨基酸序列，将重复率最高的序列化学合成多肽并进行异硫氰酸荧光素（fluorescein, FITC）标记，制备光学分子探针，初步鉴定其对NCI-H1299细胞的特异性。

**结果:**

经3轮体内筛选后噬菌体富集率是首轮的341.3倍；免疫组织化学染色显示随着筛选次数增加，肿瘤组织中结合的噬菌体不断增加，且结合量明显高于正常组织；ELISA结果显示随机挑取的30个噬菌体克隆中20个为阳性克隆，经测序后将重复率最高的序列合成多肽并命名为NSP1；四甲基偶氮唑盐比色法（methyl thiazolyl tetrazolium assay, MTT）、细胞划痕实验表明NSP1不会影响细胞增殖、迁移；流式细胞术、细胞免疫荧光结果表明NSP1可与NCI-H1299细胞特异性结合。

**结论:**

利用体内噬菌体展示技术成功得到了与肺癌NCI-H1299细胞特异性结合的多肽NSP1，为NSCLC的早期诊断及靶向治疗奠定了研究基础。

## Abstract (en)

**Background and objective:**

Non-small cell lung cancer (NSCLC) is the most common histological type of lung cancer, and one of the malignant tumor with the highest mortality. As the main part of the optical molecular imaging probe, peptide can realize the early screening and diagnosis of tumor and improve the survival rate of patients. The aim of this study was to screen the small-molecule peptide that highly binds to NSCLC NCI-H1299 cells using *in vivo* phage display technology and to identify their binding specificity by *in vitro* experiment.

**Methods:**

To prepare a tumor-bearing nude mouse model of NCI-H1299 cells, after 3 rounds of *in vivo* screening with Ph.D.-C7CTM Peptide Library, phage clones were randomly picked, using immunohistochemistry and enzyme-linked immunosorbent assay (ELISA) to identify the affinity of phage clones to NCI-H1299 cells. The positive monoclonal phages DNA was extracted and sequenced to obtain the amino acid sequence of the peptides. The peptides with the highest repetition rate was chemically synthesized and labeled with fluorescein (FITC) to prepare optical molecular probe. We preliminary identified the specificity of the probe binding to lung cancer cells by *in vitro* experiment.

**Results:**

After three rounds of *in vivo* screening, the phages enrichment rate was 341.3 times compared with the first round. Immunohistochemical staining showed that with the increase of screening times, the phages binding to tumor tissues continued to increase, and the binding amount was significantly higher than normal tissues; ELISA results showed that 20 clones among the 30 randomly selected phage clones were positive. After sequencing, the peptide with the highest repetition rate was synthesized and named NSP1; Methyl thiazolyl tetrazolium assay (MTT) and would healing assay showed that NSP1 will not affect cell proliferation and migration. Flow cytometry and immunofluorescence showed specific binding of NSP1 to NCI-H1299 cells.

**Conclusion:**

We successfully obtained the peptide NSP1 that specifically binds to lung cancer NCI-H1299 cells by *in vivo* phage display, which provide a theoretical basis for NSCLC early diagnosis and targeted therapy.

肺癌是世界上恶性肿瘤死亡的主要原因，由于其早期起病隐匿且缺乏特异性症状，因此80%以上的肺癌在确诊时已属晚期，而早期发现、诊断和靶向治疗是提高治愈率的有效途径^[1]^。目前常用的肺癌早期筛查诊断方法如低剂量螺旋计算机断层扫描（computed tomography, CT）、痰脱落细胞学检查和支气管内镜检查等特异性较低，不能及早发现肿瘤细胞在分子水平的改变^[2, 3]^。光学分子影像技术为肿瘤的早期精确诊断提供了可能^[4]^，其关键在于研发与肿瘤细胞及组织具有较高结合特异性和敏感性的靶向分子^[5]^。小分子多肽具有靶向性强、灵敏度高、组织渗透性好、易被肿瘤细胞摄取和易于修饰以增强体内稳定性和生物活性等优点，其较短的血清半衰期和较高的生物降解性使其成为许多成像方式的理想靶向探针，是肿瘤光学分子诊断和靶向治疗的重要载体^[6]^。噬菌体展示技术可在靶标未知的情况下筛选得到与其特异性结合的靶向物质，具有库容大、高通量等特点，已成为筛选肿瘤细胞或组织特异性结合肽的一个强大工具^[7]^。本研究使用噬菌体展示随机环七肽库筛选非小细胞肺癌（non-small cell lung cancer, NSCLC）细胞系NCI-H1299的靶向肽，并在体外鉴定其结合特异性，为NSCLC的分子诊断和靶向治疗提供实验基础。

## 1 材料与方法

### 1.1 实验材料

BALB/C（nu/nu）裸鼠（4周龄-5周龄，体重15 g-18 g）购自北京维通利华实验动物技术有限公司；噬菌体展示随机环七肽库试剂盒购自NEB（北京）有限公司；人NSCLC NCI-H1299细胞购自武汉普诺赛生命科技有限公司；辣根过氧化物酶标记的抗M13单克隆抗体（HRP/Anti-M13）、兔抗M13噬菌体抗体、羊抗兔-HRP标记二抗均购自美国Sigma公司；M13噬菌体单链基因组DNA快速提取试剂盒购自北京艾德莱生物科技有限公司；Matrigel基底膜基质胶购自美国BD公司。

### 1.2 噬菌体肽库体内筛选

将NCI-H1299细胞制成细胞悬液，混悬于Matrige基质胶中，接种于裸鼠双侧前肢腋窝部位，制备荷瘤裸鼠模型。麻醉裸鼠后尾静脉注射200 μL噬菌体文库，循环15 min后心脏灌注处死。将肿瘤组织称重后研磨，0.1 mol/L Glycine-Hcl（pH 2.2）洗脱10 min，1 mol/L Tris-Hcl缓冲液（pH 9.0）中和，离心后回收上清液，沉淀物用0.1%Trition X-100作用2 h释放内化噬菌体，回收洗脱产物。取10 μL用LB/IPTG/Xgal平板测定滴度，另取10 μL进行扩增纯化，用于下一轮筛选。

### 1.3 酶联免疫吸附法（enzyme-linked immunosorbent assay, ELISA）鉴定阳性噬菌体克隆

从第三轮筛选后滴度测定平板中随机挑取30个噬菌斑制备噬菌体悬液，将NCI-H1299细胞和人正常脐静脉内皮细胞HUVEC用无血清培养基37 ℃孵育1 h，4%多聚甲醛固定，3%H_2_O_2_孵育0.5 h，5%BSA封闭1 h。加入200 μL噬菌体悬液，37 ℃培养1 h-2 h。加入HRP/Anti-M13抗体37 ℃孵育1 h，TMB显色及终止液终止，酶标仪检测450 nm处OD值。将NCI-H1299的OD值设为S、HUVEC的OD值设为P，采用公式S/P，若比值大于2.5为阳性噬菌体克隆。

### 1.4 免疫组织化学染色鉴定噬菌体肽库体内分布

小鼠肿瘤组织与正常组织用4%多聚甲醛过夜固定后进行石蜡包埋、切片，脱蜡和水化。柠檬酸钠缓冲液煮沸修复20 min以暴露抗原，3%H_2_O_2_孵育10 min，5%BSA封闭0.5 h，滴加兔抗M13噬菌体抗体（1:500）于4 ℃过夜，次日滴加羊抗兔-HRP标记二抗孵育0.5 h，二氨基联苯胺（diaminobenzidine, DAB）显色后苏木素复染，脱水、中性树胶封片。待切片干燥后，用Scanscope病理切片扫描仪扫描。

### 1.5 测序及多肽合成

取ELISA结果为阳性的噬菌体悬液，按照M13噬菌体单链基因组DNA提取试剂盒提取DNA，送北京擎科生物科技有限公司测序。用DNAMAN分析软件将测序结果翻译成多肽的氨基酸序列，选择重复率最高的氨基酸序列，在NCBI/BLAST网站对其与已知蛋白质的氨基酸序列进行同源性分析，由杭州中肽生化有限公司将此序列合成靶向肽并将其命名为NSP1，合成异硫氰酸荧光素（fluorescein, FITC）标记靶向肽FITC-NSP1与荧光标记随机对照肽FITC-svNSP1。随机对照肽与靶向肽相比，氨基酸种类相同，空间结构也是环七肽，只有氨基酸组合顺序不同。

### 1.6 四甲基偶氮唑盐比色法（methyl thiazolyl tetrazolium assay, MTT）鉴定荧光探针细胞毒性

将NCI-H1299细胞悬液以1×10^4^个/mL加入96孔板中，在培养箱预培养，待细胞生长至90%以上，分别加入浓度为25 μmol/L、50 μmol/L、75 μmol/L和100 μmol/L的FITC-NSP1和FITC-svNSP1各10 μL，每组设5个复孔，5%CO_2_、37 ℃培养箱分别孵育6 h、12 h、24 h和48 h。小心吸去反应液，用PBS冲洗2遍-3遍后，每孔加5 mg/mL MTT溶液10 μL，继续培养4 h，PBS作为阴性对照。最后，用酶标仪测量450 nm处的吸光度。

### 1.7 细胞划痕实验

NCI-H1299细胞以5×10^5^个/孔接种于6孔板培养过夜，待细胞贴壁且生长至80%以上时，用200 μL无菌枪头在各孔内快速均匀划一条直线，PBS清洗后加入无血清培养基稀释的浓度为25 μmol/L的FITC-NSP1和FITC-svNSP1各500 μL，无血清培养基作为空白对照，每组设2个复孔。继续培养，动态观察并在24 h、36 h时在显微镜下进行拍照，用Image J软件计算划痕处面积以得出划痕愈合百分比。

### 1.8 流式细胞术鉴定荧光探针特异性

将FITC-NSP1和FITC-svNSP1用灭菌三蒸水溶解，调整浓度为25 μmol/L。将NSCLC细胞NCI-H1299、A549和人髓样乳腺癌细胞Bcap-37、人膀胱癌细胞EJ、人正常脐静脉内皮细胞HUVEC制成密度为1×10^6^个/mL的细胞悬液分装于EP管中，每种细胞6管，每管200 μL。每种细胞各取2管分别加入10 μL FITC-NSP1、FITC-svNSP1和等量PBS，混匀后室温下避光染色60 min。将细胞在1, 000 rpm离心5 min洗涤2次以去除游离荧光染料，过滤每管细胞后使用流式细胞仪进行分析。

### 1.9 细胞免疫荧光鉴定荧光探针与NCI-H1299细胞的结合

将生长状况良好的NCI-H1299、A549、Bcap-37、EJ细胞以1×10^5^个/孔的密度分别接种于6孔板中培养过夜，待细胞贴壁、长满单层后，用无血清培养基37 ℃孵育1 h，4%多聚甲醛固定，5%PBS-BSA封闭1 h。每板各取3孔分别加入50 μL浓度为25 μmol/L的FITC-NSP1和FITC-svNSP1避光孵育1 h。PBS清洗后加入DAPI染液室温避光孵育10 min，用荧光倒置显微镜观察拍照。

### 1.10 统计学处理

本研究采用SPSS 17.0软件进行统计学分析，计数资料采用率（%）表示，组间比较采用卡方检验；计量资料采用均数±标准差（Mean±SD）表示，组间比较采用*t*检验，以*P* < 0.05为差异有统计学意义。

## 2 结果

### 2.1 NCI-H1299细胞荷瘤裸鼠模型的建立

注射NCI-H1299细胞2周-3周后即可长出肉眼可见的瘤状肿块，逐日观察记录肿块直径，待肿块长至0.5 cm-1 cm，活动度较好，无化脓破溃即可用于体内筛选，成瘤率达90%左右（图 1A）。

**图 1:**
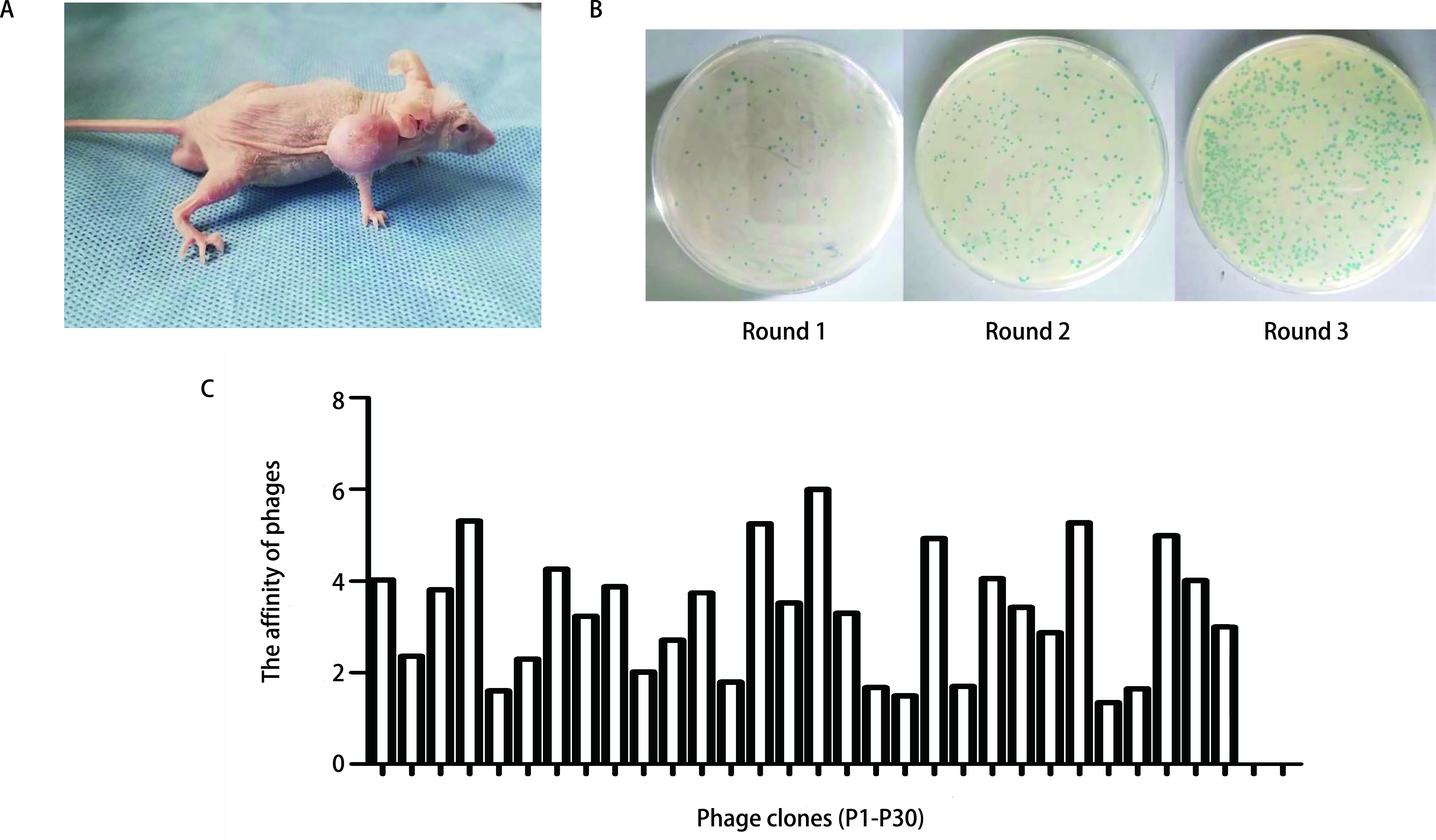
噬菌体展示技术体内筛选。A：NCI-H1299细胞荷瘤裸鼠模型；B：3轮体内筛选后肿瘤组织噬菌体滴度；C：ELISA检测单克隆噬菌体亲和力。 *In vivo* phage display. A: NCI-H1299 tumor-bearing nude mouse model; B: Tumor phage titers of three rounds of *in vivo* screening; C: ELISA detection of monoclonal phage affinity. ELISA: enzyme-linked immunosorbent assay.

### 2.2 肺癌组织特异性结合噬菌体的富集

将噬菌体展示随机环七肽库尾静脉注入荷瘤裸鼠体内，经过3轮体内亲和筛选并洗脱后进行噬菌体滴度测定，结果显示，噬菌体回收率随筛选次数增加不断提高（图 1B），到最后一轮筛选结束时，噬菌体从肿瘤组织中的回收率是首轮的341.3倍，富集效果明显（表 1）。

**表 1:** 体内筛选对特异性结合噬菌体克隆的富集效应 Enrichment effect of *in vivo* screening on specific phage clones

Panning round	Tumor quality (g)	Input phages (pfu)	Elution phages (pfu)	Recovery rate [(elution/input)/quality]	Enrichment times
1	0.48	2.0×10^10^	1.0×10^4^	1.042×10^-6^	-
2	0.42	2.0×10^10^	3.5×10^5^	4.167×10^-5^	3.999-fold
3	0.45	2.0×10^10^	3.2×10^6^	3.556×10^-4^	341.3-fold

### 2.3 肺癌细胞特异结合的阳性噬菌体克隆鉴定

利用ELISA法对第3轮筛选后随机挑选的30个噬菌体克隆进行初步鉴定，根据噬菌体克隆与NCI-H1299细胞系的结合情况，排除非特异性结合的噬菌体克隆。OD值结果显示，有20个噬菌体克隆对NCI-H1299细胞的OD_450_值显著高于对照HUVEC细胞，S/P比值大于2.5。表明以上单克隆噬菌体与NCI-H1299细胞的亲和力高，为阳性噬菌体克隆（图 1C）。

### 2.4 噬菌体在肿瘤组织及体内分布

免疫组织化学结果显示，肿瘤组织中富集的噬菌体随每一轮体内筛选的进行而增加（图 2A）。第3轮筛选后，与肿瘤组织结合的噬菌体明显多于正常组织，与肿瘤组织相比，肝脏和肾脏因血管丰富、代谢速度快，噬菌体在体内主要通过肝肾组织代谢排出，因此在肝肾组织中会滞留较多噬菌体（图 2B）。

**图 2:**
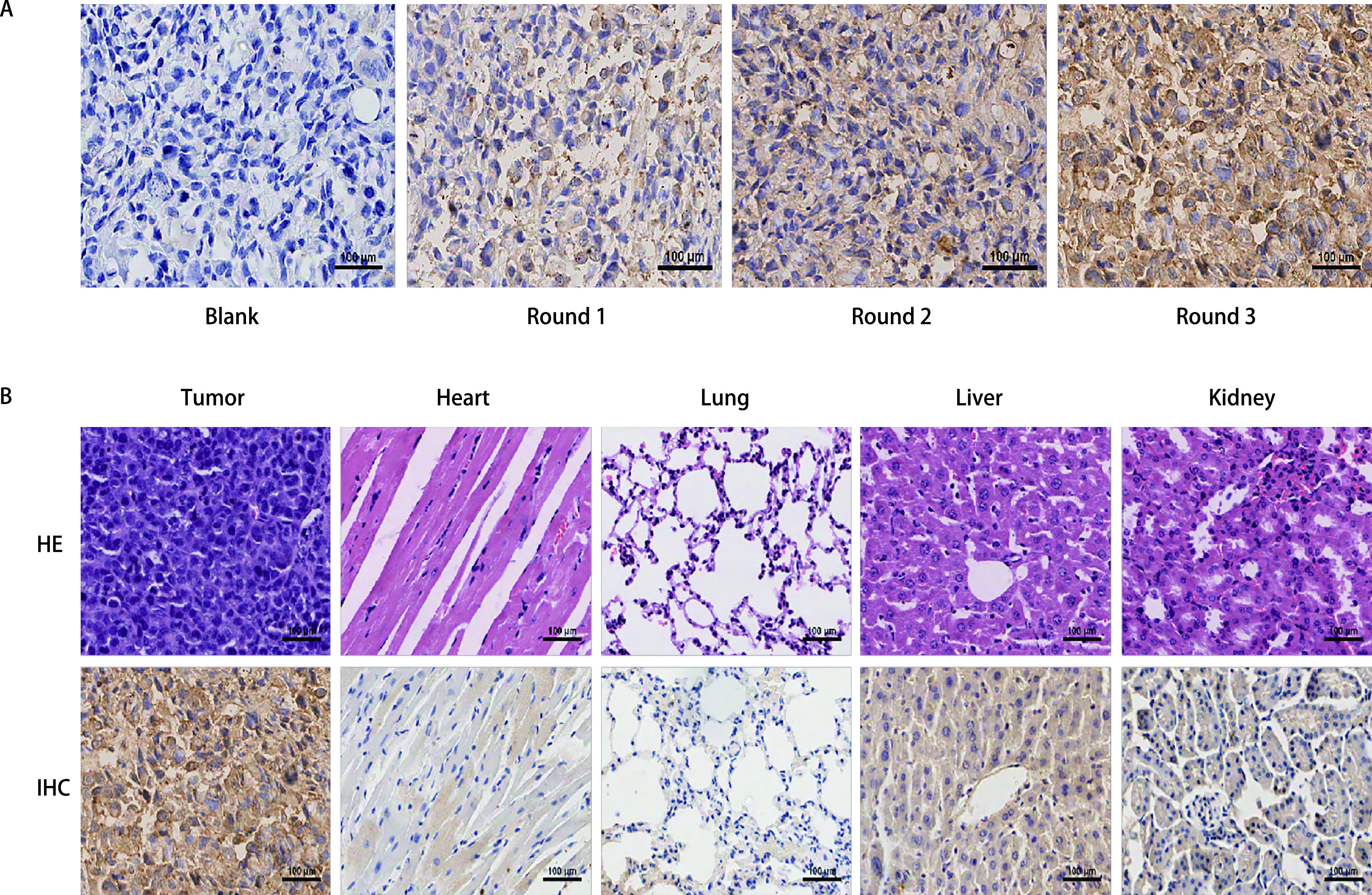
病理图片。A：三轮筛选中肿瘤组织的噬菌体含量（bar=100 *μ*m）；B：免疫组化鉴定噬菌体体内分布（bar=100 *μ*m），从左到右分别为第3轮筛选中肿瘤组织、心、肺、肝脏、肾脏的染色图，第一行为HE染色，第二行为免疫组织化学染色。 Pathological images. A: Phage content of tumor tissues in three rounds of screening (bar=100*μ*m); B: Immunohistochemical identification of phage distribution *in vivo* (bar=100 *μ*m), from left to right are the staining images of tumor tissue, heart, lung, liver, kidney in the third round of screening. The first line is HE staining, and the second line is IHC staining. IHC: immunochemistry.

### 2.5 阳性噬菌体克隆测序及多肽合成

将上述ELISA鉴定的20个阳性噬菌体克隆进行扩增并提取DNA测序，将测序结果翻译成相应的氨基酸序列，序列CTXESXGTC重复率最高（表 2），经搜索BLAST数据库未查到与已知氨基酸序列有相似性，且国内外文献均未见报道。将此序列合成靶向肽后进行后续验证实验，随机改变氨基酸组合顺序后将序列CEXGTXSTC合成随机对照肽，通过反相高效液相色谱鉴定，所合成肽纯度≥95%（图 3）。

**表 2:** 阳性单克隆噬菌体重复的氨基酸序列 Repeat amino acid sequence of positive monoclonal phages

Phage clones (S/P > 2.5)	Sequence	Repeat times
P1, P7, P14, P22	CTXESXGTC	4
P3, P12	CEXAYXYSC	2
P9, P20	CPXYKXMLC	2
P25	CTXSIXWSC	1

**图 3:**
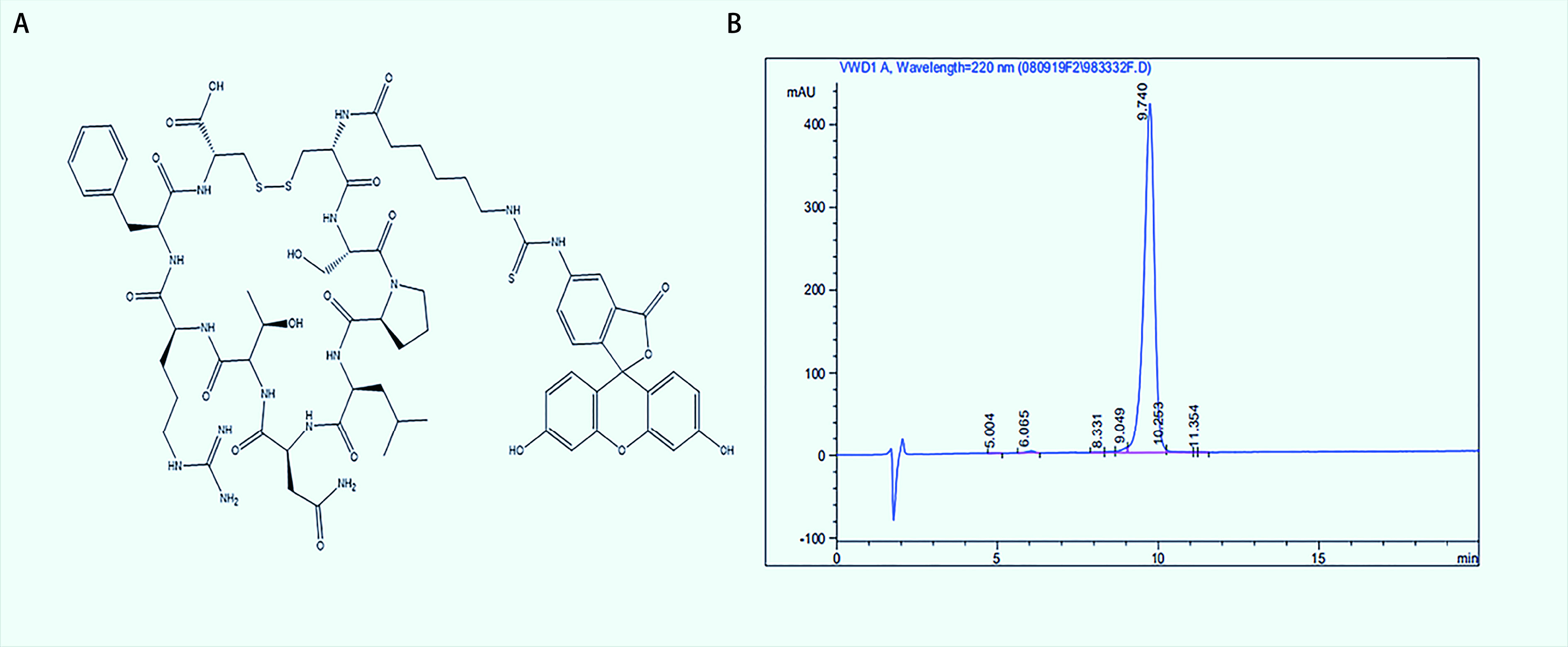
荧光探针的分子结构及纯度。A：FITC-NSP1的分子结构式；B：高效液相色谱鉴定FITC-NSP1纯度。 Structure and purity of fluorescent probe. A: FITC-NSP1 molecular structure; B: HPLC to identify the purity of FITC-NSP1. HPLC: high performance liquid chromatography; FITC: fluorescein.

### 2.6 荧光探针对细胞增殖、迁移的影响

MTT结果显示在不同的浓度下，靶向肽FITC-NSP1、对照肽FITC-svNSP1和PBS相比均未明显抑制肿瘤细胞的生长（图 4A），细胞生长曲线所示，三组的NCI-H1299细胞生长趋势一致（图 4B），各组间差异无统计学意义，表明靶向肽不会直接影响肿瘤细胞的生长（*P* > 0.05）。划痕实验结果显示，三组NCI-H1299细胞在不同时间内都表现出相同的迁移能力（图 4C），表明该荧光探针在各检测时间段均不会对NCI-H1299细胞迁移产生影响（*P* > 0.05）。

**图 4:**
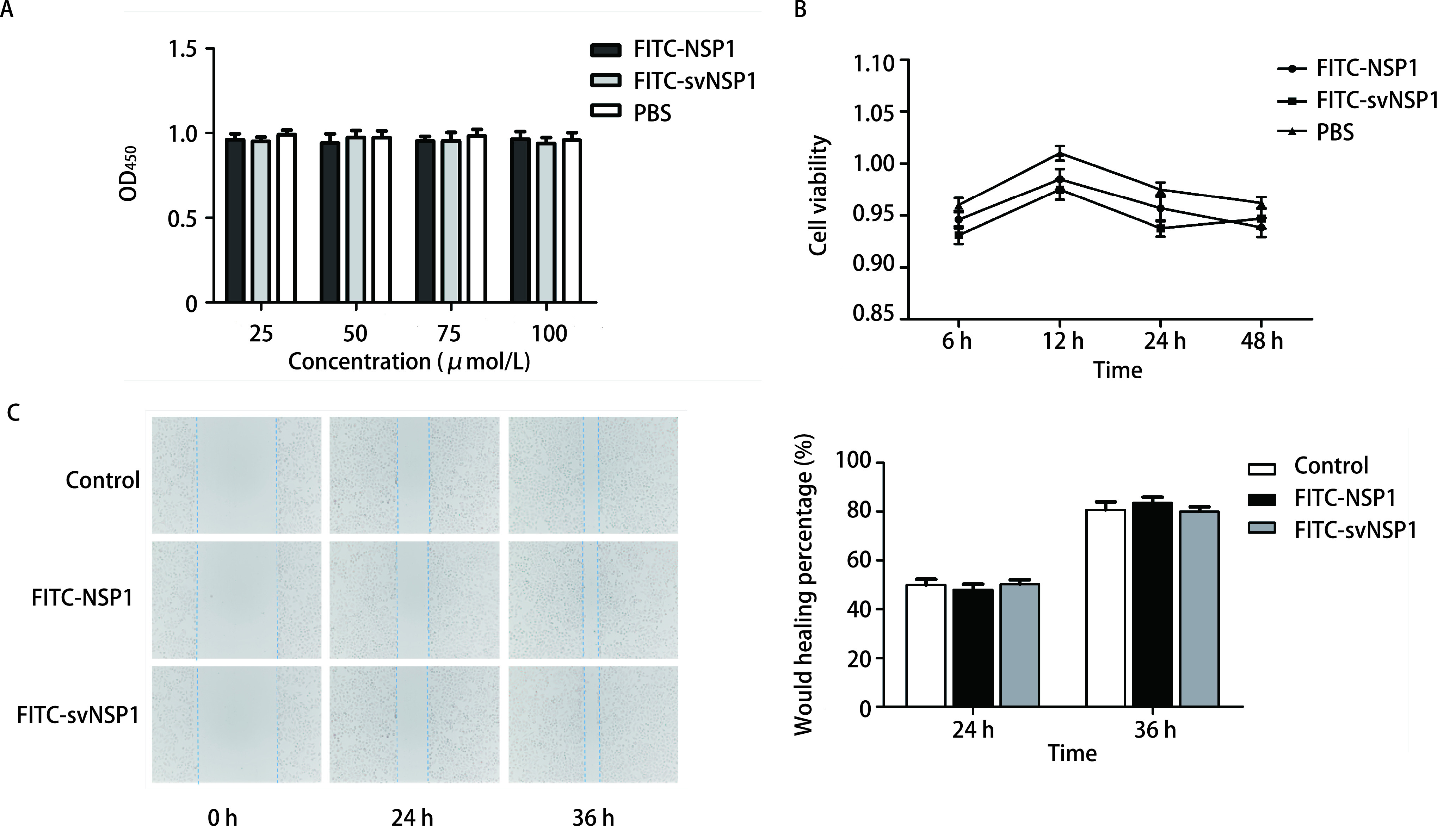
荧光探针对细胞增殖、迁移的影响。A：MTT检测不同浓度荧光探针对细胞增殖的影响；B：荧光探针作用的细胞生长曲线图；C：细胞划痕实验检测荧光探针对细胞迁移能力的影响（*P* > 0.05）。 The effect of fluorescent probe on cell proliferation and migration. A: MTT detects the effect of different concentrations of fluorescent probes on cell proliferation; B: Cell growth curve graph of fluorescent probe action; C: The migration ability of cells was measured by wound healing (*P* > 0.05). MTT: methyl thiazolyl tetrazolium assay.

### 2.7 荧光探针与NCI-H1299细胞的体外结合能力

流式细胞术结果显示，靶向肽FITC-NSP1标记NCI-H1299细胞的百分比明显高于其他细胞，且明显高于对照肽FITC-svNSP1标记的NCI-H1299细胞百分比（*P* < 0.001），表明靶向肽与NCI-H1299细胞具有特异性（图 5A、表 3）。细胞免疫荧光中，将靶向肽FITC-NSP1和对照肽FITC-svNSP1分别与NCI-H1299、A549、Bcap-37、EJ细胞孵育，观察其结合情况。将荧光显微镜下拍摄的图片用Image J软件分析荧光强度，结果显示靶向肽对NCI-H1299细胞有较高结合特异性，与人肺腺癌A549细胞也有一定的结合能力（图 5B，表 4），对照肽基本不和NCI-H1299细胞结合（图 5C），差异有统计学意义（*P* < 0.001）。

**图 5:**
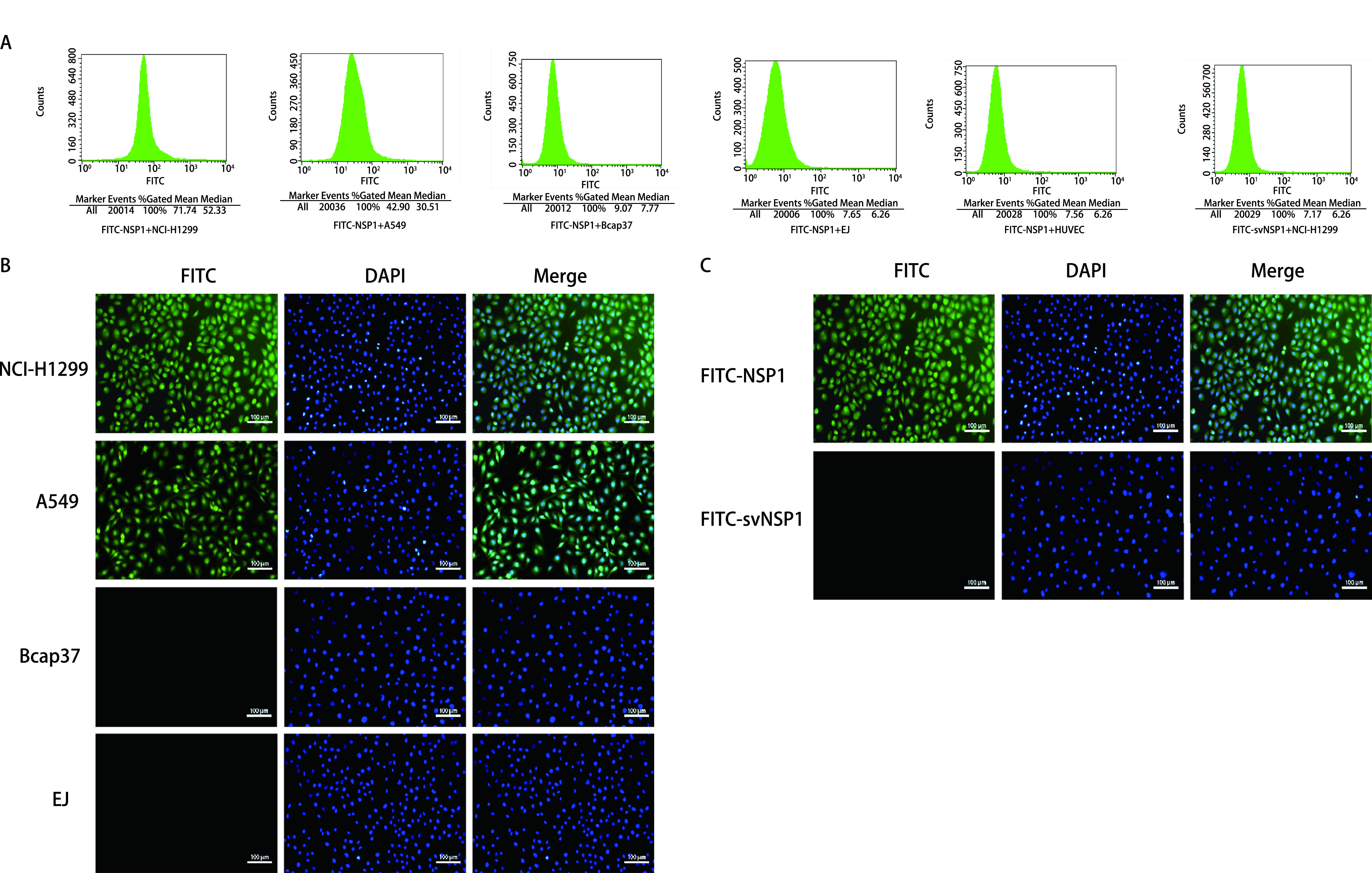
荧光探针的特异性结合鉴定。A：流式细胞术检测FITC-NSP1与FITC-svNSP1标记不同细胞的阳性百分比；B：细胞免疫荧光检测FITC-NSP1与不同细胞的结合情况；C：FITC-NSP1与FITC-svNSP1分别与NCI-H1299细胞的结合能力（bar=100 *μ*m）。 Specific binding identification of fluorescent probes. A: Flow cytometry to detect the positive percentage of FITC-NSP1 and FITC-svNSP1 labeled different cells; B: Cellular immunofluorescence to detect the binding of FITC-NSP1 to different cells; C: The binding ability of FITC-NSP1 and FITC-svNSP1 to NCI-H1299 cells, respectively (bar=100 *μ*m).

**表 3:** 流式细胞术检测荧光探针与不同细胞的结合特异性 Flow cytometry detects the binding specificity of fluorescent probes to different cells

Cell lines	Test times	Percentage of labeled cells (%)	
		FITC-NSP1	FITC-svNSP1
NCI-H1299	5	70.54±2.15^*Δ^	7.22±0.36
A549	5	41.86±1.63	-
Bcap-37	5	8.73±0.55	-
EJ	5	7.89±0.77	-
HUVEC	5	7.50±0.47	-
^*^indicates that compared with other cells, the percentage of NCI-H1299 cells labeled with FITC-NSP1 was significantly higher than other cells (*P* < 0.001). ^Δ^indicates that compared with the FITC-svNSP1, the percentage of FITC-NSP1 labeled NCI-H1299 cells increased significantly (*P* < 0.001).			

**表 4:** 细胞免疫荧光检测荧光探针与不同细胞结合的荧光强度 Cellular immunofluorescence detects the fluorescence intensity of fluorescent probes combined with different cells

Fluorescent probe	Fluorescence intensity			
	NCI-H1299	A549	Bcap-37	EJ
FITC-NSP1	103.553±8.412^*Δ^	70.267±5.493	14.152±1.095	11.721±3.509
FITC-svNSP1	9.653±0.880	12.468±2.028	7.819±0.602	6.335±1.023
^*^indicates that the fluorescence intensity of the target peptide FITC-NSP1 combined with NCI-H1299 cells is significantly higher than that of other cells (*P* < 0.001); ^Δ^indicates that the fluorescence intensity of the binding of FITC-NSP1 to NCI-H1299 cells is significantly higher than that of the FITC-svNSP1 (*P* < 0.001).				

## 3 讨论

近年来，靶向肽介导的分子影像学技术成为早期检测肿瘤病灶和提高早期诊断率的重要手段^[8]^。噬菌体展示技术是获得高亲和力高选择性多肽的有力工具，其中体内噬菌体展示技术能充分模拟人体内环境，最大程度地保持肿瘤组织和细胞表面各种配体的天然构象，在筛选过程中有各种正常组织作为背景对照，可以使肽库经过层层筛选，以最大可能性获得具备较高亲和力和特异性的短肽^[9, 10]^。迄今为止文献报道的已被成功证实可用于早期诊断的肺癌靶向肽包括：Chi等^[11]^利用体外噬菌体展示技术筛选得到肺癌H460细胞株的靶向肽HSP4，将此肽与含有抗癌药物阿霉素的脂质体联用，在动物实验中显示出较好的治疗效果，延长了肿瘤小鼠的存活期；Lee等^[12]^通过体内噬菌体展示技术筛选到人肺腺癌特异性多肽Pep-1，活体荧光成像证明该肽能选择性地结合到肿瘤组织，有望成为肺癌靶向的肿瘤显像剂。这些研究为寻找新的肺癌靶向肽打下了基础。

NCI-H1299是源自淋巴结转移的人类上皮细胞系，被广泛用于NSCLC研究，本研究利用噬菌体展示技术对NCI-H1299细胞进行3轮体内筛选，得到了特异性结合肽NSP1，并将肽偶联FITC构建小分子荧光探针。MTT和细胞划痕实验结果显示该探针没有细胞毒性，流式细胞术及细胞免疫荧光分析表明此探针与NCI-H1299细胞有较强的结合特异性和亲和力，同时与人肺腺癌细胞系A549有亲和结合，该结果提示NCI-H1299细胞表面存在多肽NSP1的特异结合位点，为之后的受体研究奠定一定的理论基础。我们下一步将选择多种肺癌细胞系进行特异性验证实验，明确NSP1荧光探针对哪一类异质性肺癌细胞群更具有特异性。

肿瘤靶向肽是当前普遍认为的比较理想的肿瘤诊断和靶向治疗载体^[13]^，具有免疫原性低、靶向特异性高及疗效显著等特点，与抗体相比更适用于肿瘤分子影像学应用^[14]^。本研究得到的NSP1可携带发光物质特异性富集在肿瘤组织进行肿瘤特异性光学成像，用于早期诊断；NSP1也可与药物偶联将细胞毒性药物特异性递送至肿瘤部位，达到治疗效果；另一方面，NSP1是环状结构，由于构象不同，环状肽比线性肽具有更好的生物活性，可以增强与目标分子的结合或受体的选择性。环结构的另一个好处是由于缺乏氨基和羧基末端，可以抵抗外肽酶的水解，更容易穿过细胞膜^[15]^，因此具有较高的体内稳定性。

本研究成功筛选到肺癌NCI-H1299细胞特异性靶向肽，并通过体外实验对多肽NSP1的特异性进行了验证，而多肽NSP1的靶向性尚需进行动物活体成像来观察示踪剂的靶向分布以从整体动物水平进一步证实。本研究为多肽NSP1在NSCLC早期诊断和靶向治疗的体外研究提供了实验基础。

## References

[1] Jin Y, Yang Y, Su Y (2019). Identification a novel clinical biomarker in early diagnosis of human non-small cell lung cancer. Glycoconj J.

[2] Inage T, Nakajima T, Yoshino I (2018). Early lung cancer detection. Clin Chest Med.

[3] Eggert JA, Palavanzadeh M, Blanton A (2017). Screening and early detection of lung cancer. Semin Oncol Nurs.

[4] Ma X, Hui H, Shang W (2015). Recent advances in optical molecular imaging and its applications in targeted drug delivery. Curr Drug Targets.

[5] Kobayashi H, Choyke PL (2011). Target-cancer-cell-specific activatable fluorescence imaging probes: rational design and *in vivo* applications. Acc Chem Res.

[6] Yavari B, Mahjub R, Saidijam M (2018). The potential use of peptides in cancer treatment. Curr Protein Pept Sci.

[7] Saw PE, Song EW (2019). Phage display screening of therapeutic peptide for cancer targeting and therapy. Protein Cell.

[8] Sun X, Li Y, Liu T (2017). Peptide-based imaging agents for cancer detection. Adv Drug Deliv Rev.

[9] Bábíčková J, Tóthová L', Boor P (2013). *In vivo* phage display--a discovery tool in molecular biomedicine. Biotechnol Adv.

[10] Newton-Northup JR, Dickerson MT, Kumar SR (2014). *In vivo* bacteriophage peptide display to tailor pharmacokinetics of biological nanoparticles. Mol Imaging Biol.

[11] Chi YH, Hsiao JK, Lin MH (2017). Lung cancer-targeting peptides with multi-subtype indication for combinational drug delivery and molecular imaging. Theranostics.

[12] Lee KJ, Lee JH, Chung HK (2016). Application of peptide displaying phage as a novel diagnostic probe for human lung adenocarcinoma. Amino Acids.

[13] Liu R, Li X, Xiao W (2017). Tumor-targeting peptides from combinatorial libraries. Adv Drug Deliv Rev.

[14] Zhao N, Qin Y, Liu H (2018). Tumor-targeting peptides: ligands for molecular imaging and therapy. Anticancer Agents Med Chem.

[15] Park SE, Sajid MI, Parang K (2019). Cyclic cell-penetrating peptides as efficient intracellular drug delivery tools. Mol Pharm.

